# (Methanol-κ*O*)-*cis*-dioxido{(4*Z*,*N*′*E*)-*N*′-[(*Z*)-4-oxido-4-phenyl­but-3-en-2-yl­idene]iso­nicotino­hydrazidato}molybdenum(VI)

**DOI:** 10.1107/S1600536813019077

**Published:** 2013-07-17

**Authors:** Sathish Kumar Kurapati

**Affiliations:** a School of Chemistry, University of Hyderabad, Gachibowli, Hyderabad, Andhra Pradesh 500 046, India

## Abstract

In the title complex, [Mo(C_16_H_13_N_3_O_2_)O_2_(CH_3_OH)], the deprotonated Schiff base (*E*)-*N*′-[(*Z*)-4-oxido-4-phenyl­but-3-en-2-yl­idene]isonicotinohydrazide coordinates in a meridional fashion through the enolate O-, imine N- and amidate O-atom donors to the Mo atom of a *cis*-[MoO_2_]^2+^ core. The sixth coordination site of molybdenum is occupied by the O atom of a methanol mol­ecule. In this complex, the NO_5_ coordination sphere adopts a distorted octa­hedral coordination geometry. The metal atom is shifted by 0.335 (1) Å from the square plane defined by the three donor atoms of the Schiff base ligand and one oxide group towards the second oxide group in the *cis* position. In the crystal, the complex forms inversion dimers through a pair of O—H⋯N hydrogen bonds involving the methanol –OH group and the pyridine N atom. Additional C—H⋯O contacts stack the mol­ecules along the *b* axis.

## Related literature

For the coordination chemistry of molybdenum, see: Arzoumanian (1998 ▶). For ligand-exchange reactions of molybdenum complexes, see: Chakravarthy & Chand (2011 ▶). For the preparation of the Schiff base, see: El-Bahnasawy & El-Meleigy (1993 ▶). For a similar type of complex, see: Jin & Li (2012 ▶). For related structures and hydrogen bonding, see: Kurapati *et al.* (2012 ▶).
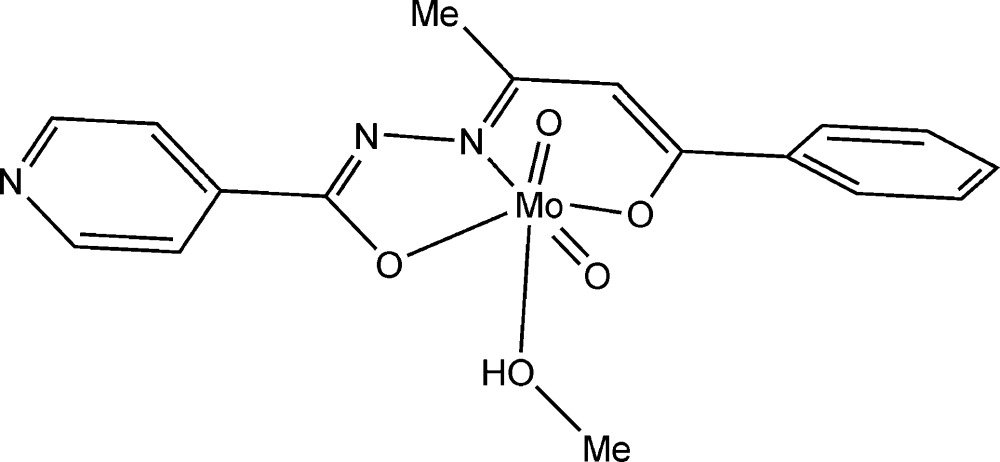


## Experimental

### 

#### Crystal data

[Mo(C_16_H_13_N_3_O_2_)O_2_(CH_4_O)]
*M* *_r_* = 439.28
Monoclinic, 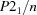

*a* = 14.3222 (9) Å
*b* = 8.4083 (5) Å
*c* = 16.0102 (10) Å
β = 113.507 (1)°
*V* = 1768.03 (19) Å^3^
*Z* = 4
Mo *K*α radiation
μ = 0.78 mm^−1^
*T* = 298 K
0.24 × 0.14 × 0.10 mm

#### Data collection

Bruker SMART CCD area-detector diffractometer
Absorption correction: multi-scan (*SADABS*; Sheldrick, 1996 ▶) *T*_min_ = 0.836, *T*_max_ = 0.927
17656 measured reflections
3474 independent reflections
3249 reflections with *I* > 2σ(*I*)
*R*_int_ = 0.026

#### Refinement

*R*[*F* ^2^ > 2σ(*F* ^2^)] = 0.026
*wR*(*F* ^2^) = 0.070
*S* = 1.07
3474 reflections
239 parameters
13 restraints
H atoms treated by a mixture of independent and constrained refinement
Δρ_max_ = 0.31 e Å^−3^
Δρ_min_ = −0.60 e Å^−3^

### 

Data collection: *SMART* (Bruker, 2002 ▶); cell refinement: *SAINT* (Bruker, 2002 ▶); data reduction: *SAINT*; program(s) used to solve structure: *SHELXTL* (Sheldrick, 2008 ▶); program(s) used to refine structure: *SHELXL97* (Sheldrick, 2008 ▶); molecular graphics: *SHELXTL*; software used to prepare material for publication: *SHELXTL*.

## Supplementary Material

Crystal structure: contains datablock(s) I, global. DOI: 10.1107/S1600536813019077/sj5344sup1.cif

Structure factors: contains datablock(s) I. DOI: 10.1107/S1600536813019077/sj5344Isup2.hkl

Additional supplementary materials:  crystallographic information; 3D view; checkCIF report

## Figures and Tables

**Table 1:** Hydrogen-bond geometry (Å, °)

*D*—H⋯*A*	*D*—H	H⋯*A*	*D*⋯*A*	*D*—H⋯*A*
O5—H5⋯N3^i^	0.88 (2)	1.84 (2)	2.695 (2)	167 (4)
C1—H1*C*⋯O2^ii^	0.96	2.63	3.554 (3)	162
C3—H3⋯O2^ii^	0.93	2.60	3.492 (2)	160
C14—H14⋯O1^iii^	0.93	2.57	3.134 (3)	119
C8—H8⋯O1^iv^	0.93	2.69	3.574 (3)	159
C7—H7⋯O5^v^	0.93	2.60	3.473 (3)	157

Symmetry codes: (i) 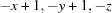; (ii) 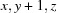; (iii) 
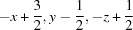
; (iv) 
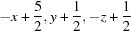
; (v) 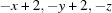.

**Fig. 1.**
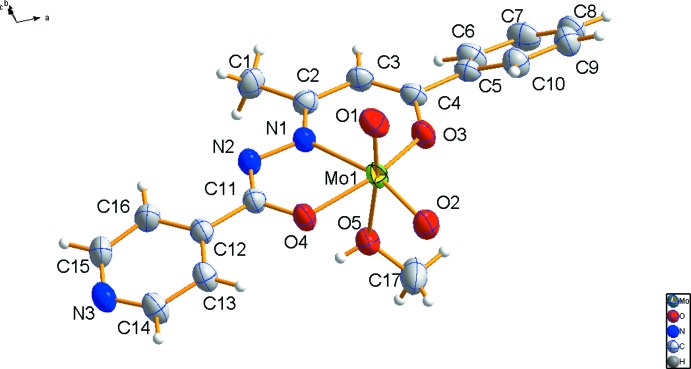
ORTEP plot of the title complex with 30% probability ellipsoids and atom-labelling scheme.

**Fig. 2.**
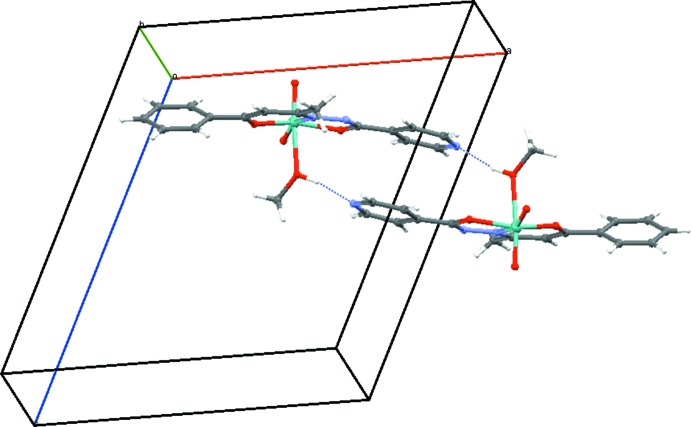
A hydrogen-bonded dimer formed through O–H···N hydrogen bonds.

**Fig. 3.**
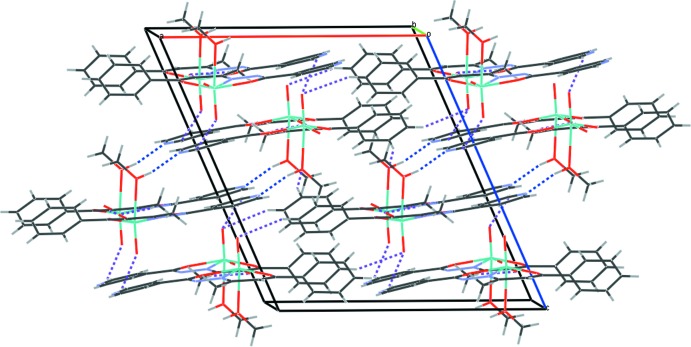
Crystal packing in the title compound viewed along the b axis.

## supplementary crystallographic information

### Comment

The coordination chemistry of *cis*-dioxomolybdenum complexes has acquired significant interest due to their catalytic ability in various organic oxidation reactions (Arzoumanian, 1998). The title complex described here was synthesized as part of our investigation into ligand exchange reactions of [MoO_2_(acac)_2_] with various Schiff-bases derived from acid hydrazides (Chakravarthy & Chand, 2011). In the present work we have used the Schiff base (*E*)-*N*'-((*Z*)-4-hydroxy-4-phenylphenylbut-3-en-2-ylidene)isonicotinohydrazide. In the title complex, the doubly deprotonated Schiff-base is coordinated to the molybdenum atom of a *cis*-[MoO_2_]^+2^ core, in a meridional fashion. The distorted octahedral NO_5_ coordination sphere around the molybdenum atom comprises two *cis* oxo groups, the ONO donor atoms of the pincer like Schiff base ligand and the O-atom of a neutral methanol molecule. The shortening of the Mo1—O1, 1.6923 (17)Å, bond distance compared to Mo1—O2, 1.7010 (14)Å, is perhaps due to the shift of the molybdenum atom from the (ONO)O square plane made up of the donor atoms of the deprotonated Schiff-base (O3,N1&O4) and O2. The Mo1 atom is displaced by 0.335 (1) Å towards O1. The Mo1–O5, 2.3649 (17) Å, and Mo1—N1, 2.2421 (16) Å, bonds are significantly longer than Mo1—O3, 1.9470 (13) Å, and Mo1—O4, 1.9951 (13) Å, which may be associated with the *trans* effect imposed by the two oxo groups. In the complex the Schiff-base is planar apart from the phenyl ring of benzoylacetone fragment which makes a dihedral angle of 36.93 (6)° with the best fit plane through the remaining non-hydrogen atoms of the Schiff base ligand.

In solid state, charge assisted intermolecular hydrogen bonding involving of methanol-OH (O5) and the pyridine-N (N3) (O—H···N) leads to formation of discrete dimeric units of the title complex (Fig.2). As a result of our investigation for other short contacts in the crystal lattice, we found five types of C—H···O contacts. In the C—H···O interactions, the H···A distances lie in the range 2.57–2.63 Å, Table 1 and together with the O—H···N hydrogen bond stack the molecules along the *b* axis.

### Experimental

The Schiff-base was prepared according to a literature method (El-Bahnasawy & El-Meleigy, 1993). The title complex was prepared following our previously reported method (Kurapati *et al.*, 2012). Solid [MoO_2_(acac)_2_] (0.1 mmol) was added to a hot methanol solution of the Schiff-base (0.1 mmol in 25 mL), and the mixture was heated on water bath for 30 minutes. The resulting bright red solution was slowly cooled to room temperature. After one day, red colored block shaped crystals were collected by filtration (Yield: 82%). One of the these crystals was used for the X-ray structural analysis.

### Refinement

All non-hydrogen atoms were refined using anisotropic thermal parameters. All hydrogen atoms bound to carbon were positioned geometrically and refined using a riding model. The H5 bound of the methanol OH group was located in a difference Fourier map and its coordinates were refined with U_eq_ = 1.5U_eq_ (O).

### Crystal data

**Table:**

[Mo(C_16_H_13_N_3_O_2_)O_2_(CH_4_O)]	*F*(000) = 888
*M**_r_* = 439.28	*D*_x_ = 1.650 Mg m^−^^3^
Monoclinic, *P*2_1_/*n*	Mo *K*α radiation, λ = 0.71073 Å
Hall symbol: -P 2yn	Cell parameters from 6758 reflections
*a* = 14.3222 (9) Å	θ = 2.4–26.0°
*b* = 8.4083 (5) Å	µ = 0.78 mm^−^^1^
*c* = 16.0102 (10) Å	*T* = 298 K
β = 113.507 (1)°	Block, red
*V* = 1768.03 (19) Å^3^	0.24 × 0.14 × 0.10 mm
*Z* = 4	

### Data collection

**Table:**

Bruker SMART CCD area-detector diffractometer	3474 independent reflections
Radiation source: fine-focus sealed tube	3249 reflections with *I* > 2σ(*I*)
Graphite monochromator	*R*_int_ = 0.026
φ and ω scans	θ_max_ = 26.0°, θ_min_ = 2.5°
Absorption correction: multi-scan (*SADABS*; Sheldrick, 1996)	*h* = −17→17
*T*_min_ = 0.836, *T*_max_ = 0.927	*k* = −10→10
17656 measured reflections	*l* = −19→19

### Refinement

**Table:**

Refinement on *F*^2^	Primary atom site location: structure-invariant direct methods
Least-squares matrix: full	Secondary atom site location: difference Fourier map
*R*[*F*^2^ > 2σ(*F*^2^)] = 0.026	Hydrogen site location: inferred from neighbouring sites
*wR*(*F*^2^) = 0.070	H atoms treated by a mixture of independent and constrained refinement
*S* = 1.07	*w* = 1/[σ^2^(*F*_o_^2^) + (0.0412*P*)^2^ + 0.6642*P*] where *P* = (*F*_o_^2^ + 2*F*_c_^2^)/3
3474 reflections	(Δ/σ)_max_ = 0.002
239 parameters	Δρ_max_ = 0.31 e Å^−^^3^
13 restraints	Δρ_min_ = −0.60 e Å^−^^3^

### Special details

**Table:**

Experimental. Selected IR data (cm^-1^): 3443 (ν_C—H_), 1610 (ν_C=N_), 935 and 906 (ν*_cis_*_-MoO2_). UV-Vis data (λ_max_ (nm) (103 *x* E (*M*^-1^ cm^-1^))): 445(5.019), 322(7.519), 272 (9.431). 1H NMR data (δ (p.p.m.) (J (Hz))): 2.507(s, 3H, H1), 6.114 (s, 1H, H3),7.737 (1.6)(d, 2H, H6&H10), 7.390(m, 3H, H7, H8&H9), 3.307 (s, 3H, H17), 7.856 (5.6) (d, 2H, H13&H16), 8.636(s, 2H, H15&H14) and 3.130 (sb, 1H, H5(Mo—OH—Me)).
Geometry. All e.s.d.'s (except the e.s.d. in the dihedral angle between two l.s. planes) are estimated using the full covariance matrix. The cell e.s.d.'s are taken into account individually in the estimation of e.s.d.'s in distances, angles and torsion angles; correlations between e.s.d.'s in cell parameters are only used when they are defined by crystal symmetry. An approximate (isotropic) treatment of cell e.s.d.'s is used for estimating e.s.d.'s involving l.s. planes.
Refinement. Refinement of *F*^2^ against ALL reflections. The weighted *R*-factor *wR* and goodness of fit *S* are based on *F*^2^, conventional *R*-factors *R* are based on *F*, with *F* set to zero for negative *F*^2^. The threshold expression of *F*^2^ > σ(*F*^2^) is used only for calculating *R*-factors(gt) *etc*. and is not relevant to the choice of reflections for refinement. *R*-factors based on *F*^2^ are statistically about twice as large as those based on *F*, and *R*- factors based on ALL data will be even larger.

### Fractional atomic coordinates and isotropic or equivalent isotropic displacement parameters (Å^2^)

**Table:**

	*x*	*y*	*z*	*U*_iso_*/*U*_eq_	
Mo1	0.897513 (11)	0.554260 (18)	0.172801 (12)	0.03495 (8)	
O2	0.93687 (11)	0.37519 (17)	0.14943 (11)	0.0500 (4)	
O4	0.75394 (10)	0.49574 (17)	0.14826 (10)	0.0395 (3)	
O3	0.99506 (10)	0.69615 (15)	0.15438 (11)	0.0437 (3)	
O1	0.94126 (13)	0.56206 (17)	0.28773 (11)	0.0506 (4)	
N3	0.38179 (14)	0.4684 (2)	0.07876 (13)	0.0473 (4)	
C12	0.58263 (15)	0.5609 (2)	0.12426 (13)	0.0362 (4)	
C3	0.93455 (16)	0.9600 (2)	0.14600 (15)	0.0390 (4)	
H3	0.9517	1.0667	0.1462	0.047*	
N1	0.80704 (11)	0.78010 (18)	0.14665 (11)	0.0353 (3)	
C4	1.00685 (14)	0.8525 (2)	0.15157 (13)	0.0354 (4)	
N2	0.70566 (12)	0.7596 (2)	0.13494 (11)	0.0394 (4)	
C11	0.68677 (14)	0.6106 (2)	0.13778 (13)	0.0354 (4)	
C2	0.83391 (16)	0.9256 (2)	0.13986 (14)	0.0364 (4)	
C7	1.2115 (2)	1.0610 (3)	0.10059 (17)	0.0514 (6)	
H7	1.2177	1.1454	0.0656	0.062*	
C1	0.76117 (19)	1.0617 (2)	0.12404 (18)	0.0496 (6)	
H1B	0.7114	1.0585	0.0624	0.074*	
H1C	0.7979	1.1603	0.1344	0.074*	
H1A	0.7277	1.0536	0.1652	0.074*	
C5	1.10730 (14)	0.8979 (2)	0.15241 (13)	0.0363 (4)	
C15	0.40852 (16)	0.6216 (3)	0.08732 (17)	0.0536 (6)	
H15	0.3579	0.6976	0.0770	0.064*	
C13	0.55630 (15)	0.4022 (3)	0.11850 (14)	0.0414 (4)	
H13	0.6055	0.3235	0.1296	0.050*	
C6	1.11753 (17)	1.0265 (3)	0.10187 (15)	0.0454 (5)	
H6	1.0613	1.0892	0.0690	0.054*	
C14	0.45571 (15)	0.3623 (3)	0.09603 (15)	0.0473 (5)	
H14	0.4388	0.2550	0.0928	0.057*	
C10	1.19297 (16)	0.8094 (3)	0.20242 (14)	0.0440 (5)	
H10	1.1872	0.7231	0.2364	0.053*	
C8	1.29577 (18)	0.9716 (3)	0.15064 (16)	0.0495 (5)	
H8	1.3586	0.9951	0.1493	0.059*	
C9	1.28662 (16)	0.8474 (3)	0.20263 (16)	0.0506 (5)	
H9	1.3438	0.7888	0.2381	0.061*	
C16	0.50678 (16)	0.6730 (3)	0.11063 (16)	0.0509 (5)	
H16	0.5222	0.7810	0.1172	0.061*	
O5	0.81999 (11)	0.57741 (18)	0.01232 (11)	0.0444 (3)	
C17	0.8607 (2)	0.5129 (4)	−0.0480 (2)	0.0728 (8)	
H17B	0.8160	0.5370	−0.1097	0.109*	
H17A	0.8670	0.3997	−0.0402	0.109*	
H17C	0.9265	0.5585	−0.0350	0.109*	
H5	0.7536 (14)	0.568 (4)	−0.010 (3)	0.109*	

### Atomic displacement parameters (Å^2^)

**Table:**

	*U*^11^	*U*^22^	*U*^33^	*U*^12^	*U*^13^	*U*^23^
Mo1	0.02528 (11)	0.02513 (11)	0.05026 (13)	−0.00067 (5)	0.01066 (8)	0.00289 (6)
O2	0.0398 (8)	0.0297 (7)	0.0768 (11)	0.0036 (6)	0.0193 (7)	0.0016 (7)
O4	0.0278 (7)	0.0312 (7)	0.0575 (9)	−0.0013 (6)	0.0150 (6)	0.0030 (6)
O3	0.0312 (7)	0.0285 (7)	0.0707 (10)	−0.0037 (5)	0.0197 (7)	0.0010 (6)
O1	0.0405 (9)	0.0504 (10)	0.0525 (9)	−0.0017 (6)	0.0096 (7)	0.0038 (6)
N3	0.0310 (9)	0.0614 (12)	0.0498 (10)	−0.0027 (8)	0.0164 (8)	−0.0002 (8)
C12	0.0290 (10)	0.0439 (11)	0.0356 (10)	−0.0022 (7)	0.0126 (8)	−0.0015 (7)
C3	0.0393 (11)	0.0275 (10)	0.0500 (12)	−0.0047 (7)	0.0175 (9)	0.0000 (8)
N1	0.0290 (7)	0.0305 (8)	0.0452 (9)	−0.0007 (6)	0.0136 (7)	−0.0005 (7)
C4	0.0342 (10)	0.0306 (9)	0.0378 (9)	−0.0063 (7)	0.0104 (8)	0.0001 (7)
N2	0.0298 (8)	0.0363 (9)	0.0519 (9)	0.0001 (7)	0.0163 (7)	−0.0013 (7)
C11	0.0293 (9)	0.0371 (10)	0.0387 (10)	0.0006 (8)	0.0121 (8)	0.0001 (8)
C2	0.0381 (11)	0.0298 (9)	0.0395 (10)	0.0017 (7)	0.0137 (8)	0.0000 (7)
C7	0.0553 (15)	0.0464 (13)	0.0554 (14)	−0.0179 (10)	0.0251 (12)	0.0006 (9)
C1	0.0479 (13)	0.0327 (11)	0.0701 (15)	0.0065 (8)	0.0256 (12)	0.0025 (9)
C5	0.0350 (10)	0.0334 (9)	0.0385 (10)	−0.0072 (8)	0.0126 (8)	−0.0039 (8)
C15	0.0347 (11)	0.0589 (15)	0.0702 (15)	0.0054 (10)	0.0243 (11)	−0.0083 (12)
C13	0.0307 (10)	0.0430 (11)	0.0480 (11)	0.0020 (8)	0.0131 (8)	0.0081 (9)
C6	0.0424 (11)	0.0399 (11)	0.0491 (12)	−0.0078 (9)	0.0132 (9)	0.0037 (9)
C14	0.0333 (10)	0.0481 (12)	0.0563 (12)	−0.0049 (9)	0.0133 (9)	0.0085 (10)
C10	0.0404 (11)	0.0413 (11)	0.0501 (11)	−0.0013 (9)	0.0179 (9)	0.0056 (9)
C8	0.0420 (12)	0.0544 (13)	0.0581 (14)	−0.0165 (10)	0.0263 (11)	−0.0135 (10)
C9	0.0374 (11)	0.0542 (13)	0.0581 (13)	0.0001 (9)	0.0170 (10)	−0.0020 (10)
C16	0.0398 (11)	0.0454 (12)	0.0692 (14)	−0.0008 (9)	0.0235 (10)	−0.0105 (11)
O5	0.0313 (7)	0.0527 (9)	0.0481 (8)	0.0000 (6)	0.0148 (7)	−0.0050 (6)
C17	0.0484 (15)	0.110 (2)	0.0628 (16)	0.0138 (16)	0.0250 (13)	−0.0120 (16)

### Geometric parameters (Å, º)

**Table:**

Mo1—O1	1.6920 (17)	C7—H7	0.9300
Mo1—O2	1.7009 (14)	C1—H1B	0.9600
Mo1—O3	1.9471 (13)	C1—H1C	0.9600
Mo1—O4	1.9950 (13)	C1—H1A	0.9600
Mo1—N1	2.2422 (16)	C5—C10	1.384 (3)
Mo1—O5	2.3656 (16)	C5—C6	1.393 (3)
O4—C11	1.326 (2)	C15—C16	1.374 (3)
O3—C4	1.328 (2)	C15—H15	0.9300
N3—C14	1.327 (3)	C13—C14	1.380 (3)
N3—C15	1.335 (3)	C13—H13	0.9300
C12—C13	1.380 (3)	C6—H6	0.9300
C12—C16	1.388 (3)	C14—H14	0.9300
C12—C11	1.479 (3)	C10—C9	1.377 (3)
C3—C4	1.350 (3)	C10—H10	0.9300
C3—C2	1.435 (3)	C8—C9	1.374 (3)
C3—H3	0.9300	C8—H8	0.9300
N1—C2	1.300 (2)	C9—H9	0.9300
N1—N2	1.399 (2)	C16—H16	0.9300
C4—C5	1.483 (3)	O5—C17	1.420 (3)
N2—C11	1.287 (3)	O5—H5	0.875 (19)
C2—C1	1.500 (3)	C17—H17B	0.9600
C7—C8	1.376 (4)	C17—H17A	0.9600
C7—C6	1.385 (3)	C17—H17C	0.9600
			
O1—Mo1—O2	105.20 (8)	C2—C1—H1C	109.5
O1—Mo1—O3	99.45 (7)	H1B—C1—H1C	109.5
O2—Mo1—O3	100.85 (6)	C2—C1—H1A	109.5
O1—Mo1—O4	97.42 (7)	H1B—C1—H1A	109.5
O2—Mo1—O4	98.30 (6)	H1C—C1—H1A	109.5
O3—Mo1—O4	150.09 (6)	C10—C5—C6	118.60 (18)
O1—Mo1—N1	96.08 (7)	C10—C5—C4	119.98 (18)
O2—Mo1—N1	157.85 (7)	C6—C5—C4	121.40 (18)
O3—Mo1—N1	81.31 (5)	N3—C15—C16	123.5 (2)
O4—Mo1—N1	72.46 (6)	N3—C15—H15	118.2
O1—Mo1—O5	171.01 (7)	C16—C15—H15	118.2
O2—Mo1—O5	83.52 (7)	C12—C13—C14	118.8 (2)
O3—Mo1—O5	80.76 (6)	C12—C13—H13	120.6
O4—Mo1—O5	78.81 (6)	C14—C13—H13	120.6
N1—Mo1—O5	75.02 (6)	C7—C6—C5	120.0 (2)
C11—O4—Mo1	118.97 (12)	C7—C6—H6	120.0
C4—O3—Mo1	136.06 (13)	C5—C6—H6	120.0
C14—N3—C15	117.08 (19)	N3—C14—C13	123.6 (2)
C13—C12—C16	118.05 (19)	N3—C14—H14	118.2
C13—C12—C11	121.02 (18)	C13—C14—H14	118.2
C16—C12—C11	120.77 (18)	C9—C10—C5	121.0 (2)
C4—C3—C2	126.36 (17)	C9—C10—H10	119.5
C4—C3—H3	116.8	C5—C10—H10	119.5
C2—C3—H3	116.8	C9—C8—C7	119.6 (2)
C2—N1—N2	115.50 (16)	C9—C8—H8	120.2
C2—N1—Mo1	130.12 (13)	C7—C8—H8	120.2
N2—N1—Mo1	114.37 (11)	C8—C9—C10	120.2 (2)
O3—C4—C3	124.06 (18)	C8—C9—H9	119.9
O3—C4—C5	112.98 (17)	C10—C9—H9	119.9
C3—C4—C5	122.94 (17)	C15—C16—C12	118.8 (2)
C11—N2—N1	109.67 (15)	C15—C16—H16	120.6
N2—C11—O4	124.17 (17)	C12—C16—H16	120.6
N2—C11—C12	118.91 (17)	C17—O5—Mo1	124.42 (15)
O4—C11—C12	116.85 (17)	C17—O5—H5	111 (3)
N1—C2—C3	120.44 (17)	Mo1—O5—H5	113 (3)
N1—C2—C1	121.57 (19)	O5—C17—H17B	109.5
C3—C2—C1	117.98 (17)	O5—C17—H17A	109.5
C8—C7—C6	120.6 (2)	H17B—C17—H17A	109.5
C8—C7—H7	119.7	O5—C17—H17C	109.5
C6—C7—H7	119.7	H17B—C17—H17C	109.5
C2—C1—H1B	109.5	H17A—C17—H17C	109.5

### Hydrogen-bond geometry (Å, º)

**Table:**

*D*—H···*A*	*D*—H	H···*A*	*D*···*A*	*D*—H···*A*
O5—H5···N3^i^	0.88 (2)	1.84 (2)	2.695 (2)	167 (4)
C1—H1*C*···O2^ii^	0.96	2.63	3.554 (3)	162
C3—H3···O2^ii^	0.93	2.60	3.492 (2)	160
C14—H14···O1^iii^	0.93	2.57	3.134 (3)	119
C8—H8···O1^iv^	0.93	2.69	3.574 (3)	159
C7—H7···O5^v^	0.93	2.60	3.473 (3)	157

Symmetry codes: (i) −*x*+1, −*y*+1, −*z*; (ii) *x*, *y*+1, *z*; (iii) −*x*+3/2, *y*−1/2, −*z*+1/2; (iv) −*x*+5/2, *y*+1/2, −*z*+1/2; (v) −*x*+2, −*y*+2, −*z*.
